# Testing a New Protocol of Nature-Based Intervention to Enhance Well-Being: A Randomized Control Trial

**DOI:** 10.3390/ijerph19073931

**Published:** 2022-03-25

**Authors:** Po Lam Ho, Tsz Wai Li, Huinan Liu, Tsun Fung Yeung, Wai Kai Hou

**Affiliations:** 1 Centre for Psychosocial Health, The Education University of Hong Kong, Hong Kong SAR, China; hopl@eduhk.hk (P.L.H.); twli@eduhk.hk (T.W.L.); s1107186@s.eduhk.hk (H.L.); ytsunfung@eduhk.hk (T.F.Y.); 2 Department of Psychology, The Education University of Hong Kong, Hong Kong SAR, China

**Keywords:** nature-based intervention, restorative environments, psychological well-being, health promotion, lunch break

## Abstract

This study aims to test the associations of nature contact with multiple dimensions of psychological functioning. A total of 90 university employees were randomly assigned to spend their lunch breaks on (1) a 30-min structured protocol of nature contact and (2) a control group for 10 consecutive weekdays. Psychological distress, psychological well-being, and work-related psychological outcomes were assessed at baseline (T1), the day after the 10-day intervention (T2), and three months after the intervention (T3). Mixed-design analyses of variance (ANOVA) were conducted. Overall, significant intervention effects were found in the structured protocol of nature contact on psychological distress, well-being outcomes, and work engagement (all *p* < 0.05), whereas the 3-month post-intervention effects were non-significant. Our study showed that nature-based activities during lunch breaks could enhance office workers’ mental health in general, but the effects could be short-lived, calling for regular nature-based programs in occupational settings.

## 1. Introduction

There is growing interest in the potential benefits of nature contact for mental health and work-related outcomes in recent years. Nature in the broadest sense can be defined as areas that contain elements of living plants, animals, geological processes, and other features of the earth [1]. Contact with nature can be achieved when an individual is exposed to the natural elements [2]. A growing body of studies has demonstrated that engaging with the natural environment such as taking a walk in nature is positively related to better emotional well-being, including lower levels of anxiety symptoms and negative affect and a higher and more sustainable positive affect [3,4,5]. Short-term exposure to urban green environments can relate to lower physiological stress responses such as cortisol secretion [6]. There is also evidence showing positive associations between nature contact and positive mental health outcomes, including life satisfaction [7], self-esteem [8], and prosocial behavior [9].

### 1.1. Active Engagement in Human–Nature Interaction

Nature contact could be classified into three forms: outdoor, indoor, and indirect [10]. Outdoor nature contact encompasses all interactions with natural elements outdoors, such as hiking and gardening [11]. Indoor nature contact refers to contact with natural components indoors, such as potted plants and landscape views from windows [12]. Indirect nature contact denotes interactions with conceptual representations of natural components indoors, such as pictures of natural landscapes and sounds of nature [13]. Among the three forms, self-reported outdoor nature contact has demonstrated the strongest positive associations with reduced stress and positive mental health, whereas indirect nature contact demonstrated the weakest associations with the outcomes [14]. Workers who had more physical access to outdoor green environments (outdoor nature contact) also demonstrated more positive workplace attitude than those who could only view greenery (indoor nature contact) [14,15].

The level of engagement has also been found to be directly proportional to health benefits. Higher levels of engagement in nature are consistently related to higher levels of positive affect and well-being across populations [16,17]. Although exposure to virtual natural environments has been found to be related to lower physiological and subjective stress responses, the absence of actual nature experience was positively associated with negative feelings and lack of a holistic sensory experience [18,19]. In a study on the stress-relieving effect of gardening [20], 30 participants were induced with stress through performing a demanding Stroop task and were then randomly assigned to either a 30-min outdoor gardening or indoor reading. While both gardening and reading activities were related to subsequently reduced cortisol levels and increased positive emotions, the changes were significantly larger among the gardening group. Recent studies have also highlighted the need to feel psychologically connected to nature in order to foster positive outcomes [16]. This kind of emotional bond with nature is referred to as nature connectedness, which has been found to be positively associated with multiple indicators of well-being, including higher positive affect, subjective happiness, life satisfaction, and vitality [21]. Increasing the level of engagement as well as nature connectedness could be effective for eliciting the positive mental health impact of human–nature interactions.

### 1.2. Multiple Sensory Processes

One possible way to enhance engagement is through multi-sensory stimulation. However, most research on nature’s health benefits has focused on a single sensory pathway of nature contact, especially the visual sense, with low levels of engagement [22]. For example, urban park views compared to urban roadway views were associated with better physiological stress recovery, including lower levels of skin conductance, slower heart rate, improved attention, and higher perceived restorativeness [23].

Humans are multisensory, and it is the multiple contents of nature that contribute to better health [18,24]. Humans perceive more than visual stimuli in real environments and formulate a holistic impression on nature by integrating sensations from multiple sensory inputs. Previous studies found that sensory stimulations such as sounds, smells, or temperature together with vision are needed to induce a realistic experience of a simulated environment [18,25]. Natural sounds such as birdsongs could facilitate the recovery process of the sympathetic nervous system [26] and were positively related to perceived restoration, denoting the process through which psychological and physiological recoveries are induced by particular environments or environmental compositions [27] and attention recovery [28]. Smells can function as a trigger or symbol for nature, and the natural scents of plants and flowers can be related to increased positive emotions of calmness, alertness, and others [29]. Natural scents such as bee wax and summer air have been found to predict higher levels of happiness [30]. Tastes of nature may refer to natural food consumption and gardening [24]. For example, non-commercial community gardening was found to improve participants’ self-esteem and positive affect relative to non-participants without gardening activities [31].

Finally, hands-on activities of playing and learning that involve physical touch of natural elements have been found to be associated with higher levels of confidence, motivation, and focused attention among children [32] and positive psychological well-being [17]. Nature-based physical recreational activities such as hiking, walking, hunting, and fishing were consistently positively associated with higher levels of life satisfaction, subjective vitality, positive affect, and lower levels of depressive symptoms [33].

### 1.3. The Importance of Nature Contact in the Workplace

The workplace has been recognized as one of the most significant social contexts for addressing mental health issues and promoting mental wellness [34,35]. Despite the general consensus on the positive impacts of contact with nature on human mental health [3,4,5], the potential benefits of contact with nature in the workplace are still understudied. Work-related stress refers to the pressure or demands that employees face in the work environment, such as work overburden, poor employee–employer relationships, and a lack of support and job autonomy [36]. Workplace stress is considered as one of the most important factors affecting employees’ health and well-being and effective organizational functioning across the globe [37]. On the contrary, workplace happiness and well-being have been found to be beneficial for organizations, including greater work productivity [38], increased daily creativity at work [39], higher commitment, and lower turnover [40]. Since prolonged exposure to workplace stress could bring deleterious effects to workers as well as organizations, effective interventions (i.e., nature-based interventions) are needed to promote well-being and prevent stress.

### 1.4. The Present Study

The aim of the present study is to investigate the effectiveness of a nature-based intervention on adaptive psychological functioning. Most previous studies focused on the passive reception of sensory information from the natural environment, particularly visual objects in nature. In fact, both the level of engagement and the number of sensory processes should be taken into account for assessing the health benefits of nature contact and designing nature-based mental health interventions. In particular, we administered the interventions among full-time office workers and assessed different dimensions of psychological and work-related outcomes.

We compared a structured protocol with a high level of nature engagement and involvement of multiple sensory processes with a waitlist control group. We expected that participants in the structured protocol of nature contact will report higher levels of mental health and more positive work-related functioning immediately and three months after the interventions.

## 2. Materials and Methods

### 2.1. Participants and Procedure

This study is a randomized controlled trial that was registered at www.clinicaltrials.gov (NCT05276245). Overall, 107 employees were recruited from The Education University of Hong Kong. Recruitment advertisements were disseminated to all administrative staff and research staff by email and posted on the university intranet. Inclusion criteria were (1) 18 years or older, (2) Cantonese speaking, and (3) full-time employment. Data were collected in two periods, November 2019 to May 2020 and February to June 2021. Eligible participants were randomly allocated to a (1) structured protocol of nature contact and a (2) control group. Four participants from the intervention group and three participants from the control group were excluded because they did not complete the required tasks. The final sample consisted of 90 eligible participants (age 22–60 years; M = 34.76, SD = 9.310). A total of 48 participants took part in the structured protocol of nature contact (2 males, 46 females) and 42 in the waitlist control condition (8 males, 34 females).

Ethical approval was obtained from the Human Research Ethics Committee of The Education University of Hong Kong. The final sample of 90 eligible participants was asked to give informed consent after being fully apprised of the study aim and design. All participants were arranged to spend their lunch break on a (1) 30-min structured protocol of nature contact or a (2) control group (usual lunch break) on 10 consecutive weekdays. Before the experiment started, all participants were told to eat their lunch in office. Then, participants who were randomly assigned to the intervention group were instructed to leave their offices and spend their lunch break on a 30-min structured protocol of nature contact, whereas those who were randomly assigned to the waitlist control group were instructed to stay at the office to spend their lunch break. In addition, the waitlist control group was told to wait for at least three months before they took part in the structured protocol of nature contact. All participants were instructed to fill out an online questionnaire before the intervention (T1), the day after the 10-day intervention (T2), and 3 months after the intervention (T3).

Several procedures were taken to ensure commitment and reduce non-response among participants [41]. First, each participant received an email outlining the timeline and design of the entire research process and the date of each intervention day. Second, remuneration was provided for participants who had completed the whole intervention (HKD550–650, ~USD70–82).

### 2.2. Experimental Conditions

#### Structured Protocol of Nature Contact (Program Group)

This structured protocol was a 10-day nature-based program with a high level of nature engagement and involvement of multiple sensory processes. The program was held in lunch breaks between 12:00 noon and 2:00 p.m. Each session consisted of 30 min in the Eco Garden of the Education University of Hong Kong. Activities of this program included walking, ecological photography, sketching butterflies, planting vegetables, drinking herbal tea, observing birds, and taking a nap in nature. Each activity corresponded to specific sensory pathways. Table A1 describes the activities for each sense. For example, butterfly sketching is focused on the use of visual senses, whereas drinking herbal tea involves senses of taste, smell, and touch. Trainers with master’s degrees in environmental education and rich experience in guiding ecological tours provided instructions and guidance to participants during the activities. Participants were also asked to pay attention to their surroundings and focus on their five senses during participation.

### 2.3. Measures

#### 2.3.1. Demographics

A standardized proforma was used to obtain participants’ demographic information, including age, gender (male or female), marital status (unmarried, divorced/widowed, or married/cohabitating), education level (no formal education, primary, secondary, or tertiary/above), monthly household income, (≤HKD 9999, HKD 10,000–19,999, HKD 20,000–29,999, HKD 30,000–39,999, HKD 40,000–49,999, HKD 50,000–59,999, ≥HKD 60,000), and medical histories of psychiatric conditions.

#### 2.3.2. Psychological Distress

Anxiety symptoms. The Chinese version of the 7-item Generalized Anxiety Disorder (GAD-7) was used to measure anxiety symptoms at all time points [42]. Participants rated each item on a 4-point scale ranging from 0 (not at all) to 3 (nearly every day) based on their experience in the past two weeks. Higher scores indicated greater severity of anxiety symptoms (range = 0–21). The measure showed high internal consistency (Cronbach’s α = 0.93) and scores were consistently inversely correlated with self-rated physical and mental health across different populations, including Chinese [43]. Alphas were >0.90 in all three administrations in the current study.

Depressive symptoms. Depressive symptoms were measured using the Chinese version of the Patient Health Questionnaire-9 (PHQ-9) [44] at T1, T2, and T3, respectively. Participants rated each item on a 4-point scale as in GAD-7. Higher scores indicate the greatest severity of the depressive symptoms (range 0–27). The Chinese version has demonstrated high internal consistency (α > 0.80) among Chinese subjects [44]. In this study, the Cronbach’s alphas for the three administrations were 0.83, 0.87, and 0.85, respectively.

Perceived stress. Perceived stress was assessed at all time points using the Chinese version of the 10-item Perceived Stress Scale (PSS-10) [45]. Participants reported their subjective stress in the past two weeks on a 5-point scale (0 = never, 4 = very often). Total scores ranged from 0 to 40, with higher scores indicating higher levels of perceived stress [46]. The scale was found to be reliable (α = 0.86) and valid among Chinese adults [45]. Alphas for the scale were 0.83, 0.86, and 0.85, respectively, in the current three administrations.

#### 2.3.3. Psychological Well-Being

Subjective well-being. The WHO-Five Well-Being Index (WHO-5) consisted of five positively worded items [47]. Participants were asked to report the presence of the five positive mental states in the past two weeks on a 6-point scale ranging from 0 (at no time) to 5 (all of the time). Alphas for the scale were >0.90 in the current three administrations.

Satisfaction with life. Satisfaction with Life Scale (SWLS) [48] was used to assess participants’ well-being with regard to satisfaction and fulfillment of their current state on a 4-point scale (1 = strongly disagree, 4 = strongly agree). The five items were summed, with higher scores indicating higher life satisfaction (range = 5–20). Previous studies showed that the scale is a reliable and valid measure of subjective well-being among the general population [49] and has demonstrated high internal consistency (α = 0.90) across studies among Chinese [50]. Alphas for the scale were 0.86, 0.86, and 0.83, respectively, in the current three administrations.

Positive affect. The 10-item positive affect subscale of Chinese Affect Scale (CAS-PA) [51] assessed the frequency of positive emotional experiences in the past two weeks on a 5-point scale (0 = not at all, 4 = all the time). The scale demonstrated high internal consistency (α > 0.80) and valid correlations with relevant psychosocial measures across different Chinese populations [52]. Alphas for the scale were 0.89, 0.94, and 0.84, respectively, in the current three administrations.

#### 2.3.4. Work-Related Outcomes

Burnout. Burnout was measured using the Chinese version of the 14-item Maslach Burnout Inventory-General Survey (MBI-GS) [53] at all time points. Participants rated how often they had different experiences of burnout in the past two weeks on a 7-point scale (0 = never, 6 = always). The scale consisted of five questions on emotional exhaustion, four on cynicism, and five on professional efficacy. The Chinese version has demonstrated high internal consistency (α > 0.85) among Chinese [54]. Alphas for the scale were >0.70 in the current three administrations.

Work engagement. The Chinese version of the 17-item Utrecht Work Engagement Scale (UWES) [55] was used to assess vigor, dedication, and absorption at work at all time points. Participants indicated the frequency of these experiences at work in the past two weeks on a 7-point scale (0 = never, 6 = always). Previous research has reported high internal consistency (α > 0.80) of the scale across studies in Chinese [56]. Alphas for the scale were >0.90 in the present study.

### 2.4. Statistical Analysis

All analyses were conducted using IBM SPSS Statistics version 26.0 (Armonk, NY, USA). A two-tailed significance level of *p* < 0.05 was used to determine statistical significance. To begin with, we tested whether there were differences in demographics or between dependent variables at baseline. Repeated-measures Analyses of Variance (ANOVAs) were then used to analyze the changes in each outcome across time in the two different groups. Group (i.e., program and control) was the between-subject factor, whereas timepoint (i.e., T1, T2, T3) was the within-subject factor. We assessed both the main effects of group and timepoint and the group × timepoint interaction effects. Changes in dependent variables between T1 and T2 were considered as “intervention effects”, whereas changes between T1 and T3 as “post-intervention effects”. We also used one-way ANOVA to assess whether there were significant differences between the program group and the control group in each outcome at T2 and T3 separately.

## 3. Results

### 3.1. Sample and Descriptive Statistics

The 90 participants ranged in age between 22 and 60 years (M = 34.76, SD = 9.310); 80 (88.9%) were female. A total of 84 (93.3%) reported receiving tertiary education or above, and 6 (6.7%) received secondary education. There were no differences in the main sample characteristics between the intervention groups and the control group (*p* > 0.05), except for the gender ratio (Table 1). In addition, group differences in all outcomes at the baseline were identified only in work engagement (Table A2).

### 3.2. Psychological Distress

Main effects of group was significant on depressive symptoms (*F*(1, 88) = 5.832, *p* = 0.018) and perceived stress (*F*(1, 88) = 7.213, *p* = 0.009), whereas there were significant main effects of timepoint on anxiety symptoms (*F*(2, 176) = 3.628, *p* = 0.032) and perceived stress (*F*(2, 176) = 7.754, *p* = 0.001), as well as marginally significant on depressive symptoms (*F*(2, 176) = 3.019, *p* = 0.055) (Table 2). The group × timepoint interaction effects were significant on anxiety symptoms and perceived stress (all *p* < 0.05, see Table 2). In particular, the program group experienced a steep decline in anxiety symptoms and perceived stress at T2 compared to T1, but the levels returned to T1 levels at T3, all *p* < 0.01. For depressive symptoms, the program group also showed a sharp decrease from T1 to T2 (*p* = 0.006), but the effect remained relatively stable at T3 (*p* = 0.211) (Table 3).

Although neither main effects of timepoint on depressive symptoms nor main effects of group on anxiety were observed in the repeated measures ANOVAs, the one-way ANOVA test showed that all three variables of psychological distress were significantly lower in the program group than the control group at T2 (all *p* < 0.01). In addition, the program group also demonstrated significant lower level of perceived stress than the control group at T3 (*p* = 0.019, see Table 4).

### 3.3. Psychological Well-Being

Repeated measures ANOVAs of well-being-related outcomes (i.e., subjective well-being, satisfaction with life, and positive affect) showed that the main effects of group were non-significant, all *p* > 0.05 (Table 2). There were significant main effects of timepoint on subjective well-being (*F*(2, 176) = 6.825, *p* = 0.002), satisfaction with life (*F*(2, 176) = 5.865, *p* = 0.041), and positive affect (*F*(2, 176) = 7.332, *p* = 0.001). In addition, the group × timepoint interaction effects were significant for subjective well-being (*F*(2, 176) = 6.067, *p* = 0.004) and positive affect (*F*(2, 176) = 4.577, *p* = 0.035). Overall, subjective well-being, satisfaction with life, and positive affect were consistently higher at T2 compared to T1 (*p* < 0.01). Post-intervention effects (i.e., changes between T1 and T3) was found only on satisfaction with life, *p* = 0.018. Regarding the group × timepoint interaction, the program group experienced an increase in positive affect at T2 relative to T1 (*p* = 0.000), but there was a decline in positive affect at T3 compared to T2 (*p* = 0.016). The results are summarized in Table 3.

Although no main effects of group on all outcomes were found across timepoints, additional one-way test showed that subjective well-being was significantly higher in the program group than the control group at T2 (*p* = 0.005, see Table 4).

### 3.4. Work-Related Outcomes

Significant main effects of group was only identified in work engagement, *F*(1, 88) = 9.035, *p* = 0.003, whereas significant main effects of timepoint was found in burnout only, *F*(2, 176) = 4.630, *p* = 0.011. No significant group × timepoint interaction effects could be found across both work-related outcomes, all *p* > 0.05, see Table 2. Burnout significantly increased from T1 to T2 among the control group (*p* = 0.015, see Table 3). In order to obtain a full picture of the changes in burnout levels between groups across timepoints, we analyzed the three subscales of MBI-GS separately. The subscale of professional efficacy was significantly higher at T2 compared to T1 in the program group (*p* = 0.011), whereas scores of cynicism were significantly increased at T2 compared to T1 in the control group (*p* = 0.011), see Table 3. On the other hand, the scores of emotional exhaustion and cynicism were significantly lower in the program group than the control group at T2, all *p* < 0.05 (see Table 4).

For work engagement, the main effects of group were significant, *F*(1, 88) = 9.035, *p* = 0.003, whereas the main effects of timepoint (*F*(2, 176) = 2.451, *p* = 0.094) and the group × timepoint interaction effects (*F*(2, 176) = 1.984, *p* = 0.145) were both non-significant. The one-way ANOVA test showed that work engagement was higher in the program group than the control group at both T2 (*p* = 0.001) and T3 (*p* = 0.017). The results are summarized in Table 4.

## 4. Discussion

In this study, a randomized controlled trial was designed to examine the effects of contact with nature on employees’ mental health and work-related functioning. We compared (1) a structured protocol with a high level of nature engagement and involvement of multiple sensory processes (the program group) with (2) a control group across three timepoints. As hypothesized, notable intervention effects (i.e., T1–T2 changes) on positive mental health and work-related functioning (except for burnout) were identified in the program group, consistent with previous evidence on the positive associations between nature contact and mental health [4,5]. In addition, a significant group difference between the program group and the control group was also found across most of the outcomes. However, contrary to our expectations, we did not find consistent post-intervention effects (i.e., T1–T3 change) across most of the outcomes (except for satisfaction with life) in the program group.

Our findings were consistent with the positive link between multisensory immersive environments and greater perceived stress recovery [57] and satisfaction with life [7] in previous studies. Furthermore, we found that the program group reported lower burnout in the dimensions of emotional exhaustion and cynicism, but greater work engagement in terms of all dimensions immediately after the intervention. The present findings are compatible with correlational research, which showed that participants who have higher exposure to nature reported significantly lower burnout in the dimension of cynicism but greater work engagement in the dimensions of vigor and dedication than the participants with lower exposure to nature [58]. The findings are also parallel to recent longitudinal studies on employees, which revealed that nature-based physical activity during leisure time contributed to perceived vitality [59].

The major difference between our study and previous studies (e.g., [6,22,23]) is that our study lied within more involvement and goal-oriented ways of interacting with the nature within the program group. In fact, it has been suggested that engaging in goal-oriented nature activities may encourage people to interact with nature on a regular basis and to spend longer time in natural settings [20,60]. Our research provided further evidence that the active involvement of multiple sensations in nature-based activities could be linked with higher levels of well-being and more positive work-related functioning.

Contrary to the significant time effects on mental health outcomes in the control groups in previous similar studies [41,61], outcomes on psychological distress, psychological well-being, and work engagement did not change over time in the control group, suggesting more confidence in attributing the group differences in the outcomes to the effect of the interventions. Another overall finding is that the levels of the outcomes at T3 became similar to those at T1. That the intervention effect (i.e., T2) on all outcomes lasted only for the intervention period in the program group was consistent with some previous evidence [61]. Therefore, the mental health benefits of nature-based intervention could be significant immediately, but the benefits could diminish over time.

Concerning the nonsignificant difference in burnout scores in the program group between T1 and T2, one possible explanation is that burnout could be the result of relatively longer-term experiences that cannot be easily changed within a short period of time. Burnout has been understood as a response to chronic emotional stressors at work and a slow process of progressive exhaustion [62]. We nonetheless found an increase in burnout from T1 to T2 only in the control group but not in the intervention group, suggesting that although the nature contact activities might not reduce office workers’ burnout, those activities could keep their burnout stable over the intervention period. The broaden-and-build model [63] proposed that experiencing positive emotions could broaden people’s personal resources, which may protect people against the occurrence of burnout in time. It is possible that our intervention buffers the impact of burnout on workers through its effects on reducing psychological distress and increasing positive affect. Longer exposure to the intervention and follow-up periods are needed to discern the interrelations among changes in distress, well-being, and work-related functioning.

Taking together the significant effects of the structured program group on reducing employees’ distress and enhancing employees’ well-being throughout the working day, it is suggested that positively experienced nature-based activities during lunch breaks may help employees cope with job stress and maintain their well-being throughout the day. Our findings also shed light on the importance of instilling nature contact in work settings, especially in urban cities such as Hong Kong. Workplace stress affects not only employees’ health and well-being but also organizational functioning [37]. In addition, the design and construction of an accessible and user-friendly greenspace or eco-garden should be taken into consideration.

### Limitations

Several limitations should be considered in evaluating the current findings. First, the intervention period in this study was short. As mentioned above, we did not find significant results for trait-like outcomes such as burnout. In addition, the intervention effects on most of the outcomes were short-lived. Future randomized control trials could be designed with longer intervention and follow-up periods. Second, the sample in our study was mainly women with high education levels. Similar intervention effects have been observed in organizational research that was predominantly conducted among highly educated women [6,41]. External validity of our research findings could be limited by the sample bias, discounting the generalizability of the findings to other employment groups with men, manual labor, and low education requirements. A sample with more heterogeneous groups of employees might be needed in future studies.

## 5. Conclusions

Notwithstanding the limitations, this randomized controlled trial was designed with the concepts from environmental psychology and organizational psychology to investigate the effectiveness of a structured protocol of nature contact in enhancing mental health and work-related functioning. The consistent overall effect on psychological distress, well-being, and work engagement immediately after the intervention period suggests a consistent positive association between active contact with nature and mental health. To achieve more sustainable benefits of nature-based interventions, longer interventions and follow-up periods are needed. It is also suggested that landscape design, planning, and management should pay more attention to the need for contact with nature in work settings.

## Figures and Tables

**Table 1:** Background characteristics of the participants at baseline.

	All Groups	Program	Control	*p*-Value
Sample size	*n* = 90	*n* = 48	*n* = 42	
Mean Age	34.76	34.51	35.05	0.786
Gender % female	88.9	51.1	37.8	0.025
Marital status %				0.289
Unmarried	60.0	28.9	31.1	
Married/ Cohabitating	35.6	22.2	13.3	
Divorced	3.3	1.1	2.2	
Widowed	1.1	1.1	0	
Education level %				0.867
No formal education	0	0	0	
Primary	0	0	0	
Secondary	6.7	3.3	3.3	
Tertiary or above	93.3	50.0	43.3	
Monthly household income (HKD) %				0.641
$9999 or below	0	0	0	
$10,000–$19,999	6.7	4.4	2.2	
$20,000–$29,999	11.1	3.3	7.8	
$30,000–$39,999	26.7	13.3	13.3	
$40,000–$49,999	13.3	8.9	4.4	
$50,000–$59,999	16.7	10.0	6.7	
$60,000 or above	25.6	13.3	12.2	
Have received psychological treatment %	14.4	8.9	5.6	0.527

**Table 2:** Results of repeated measures ANOVAs for the effects between groups (program group and control group), across timepoints (T1, T2, and T3), and for group × timepoint interaction.

	*F*-Value (*p*-Value)		
	Timepoint Effect	Group Effect	Group × Timepoint Effect
Anxiety symptoms (GAD-7)	3.628 (0.032) *	2.896 (0.092)	3.224 (0.046) *
Depressive symptoms (PHQ-9)	3.019 (0.055)	5.832 (0.018) *	2.278 (0.110)
Perceived stress (PSS-10)	7.754 (0.001) ***	7.213 (0.009) **	4.698 (0.011) *
Subjective well-being (WHO-5)	6.825 (0.002) **	1.039 (0.311)	6.067 (0.004) **
Satisfaction with life (SWLS)	3.257 (0.041) *	0.249 (0.619)	2.450 (0.089)
Positive affect (CAS-PA)	7.332 (0.001) ***	0.002 (0.964)	4.577 (0.015) *
Burnout (MBIGS)	4.630 (0.011) *	2.293 (0.134)	0.568 (0.568)
Emotional exhaustion (MBIGS_EE)	1.918 (0.154)	5.041 (0.027) *	1.231 (0.293)
Cynicism (MBIGS_CY)	2.031 (0.158)	3.154 (0.079)	2.278 (0.107)
Professional efficacy (MBIGS_PE)	3.006 (0.086)	2.767 (0.100)	0.495 (0.589)
Work Engagement (UWES)	2.451 (0.094)	9.035 (0.003) **	1.984 (0.145)
Vigor (UWES_VIG)	1.537 (0.219)	8.560 (0.004) **	2.772 (0.068)
Dedication (UWES_DEDI)	1.275 (0.281)	6.403 (0.013) *	1.689 (0.190)
Absorption (UWES_ABS)	3.504 (0.037)*	9.592 (0.003) **	0.910 (0.397)

* *p* < 0.05, ** *p* < 0.01, *** *p* < 0.001. GAD-7, 7-item Generalized Anxiety Disorder; PHQ-9, Patient Health Questionnaire-9; PSS-10, 10-item Perceived Stress Scale; WHO-5, WHO-Five Well-being Index; SWLS, Satisfaction with Life Scale; CAS-PA, 10-item positive affect subscale of Chinese Affect Scale; MBIGS, 14-item Maslach Burnout Inventory-General Survey; MBIGS_EE, 14-item Maslach Burnout Inventory-Emotional exhaustion subscale; MBIGS_CY, 14-item Maslach Burnout Inventory-Cynicism subscale; MBIGS_PE, 14-item Maslach Burnout Inventory-Professional efficacy subscale; UWES, 17-item Utrecht Work Engagement Scale; UWES_VIG, 17-item Utrecht Work Engagement Scale-Vigor subscale; UWES_DEDI, 17-item Utrecht Work Engagement Scale-Dedication subscale; UWES_ABS, 17-item Utrecht Work Engagement Scale-Absorption subscale.

**Table 3:** Mean difference of all outcomes within groups (program and control) across timepoints (T1, T2, and T3).

Outcomes	Mean Difference (*I–J*) ^a^					
	T2 vs. T1		T3 vs. T2		T3 vs. T1	
	Program	Control	Program	Control	Program	Control
GAD-7	−1.542 **	−0.667	1.688 **	−0.396	0.146	−1.062
PHQ-9	−1.542 **	−0.190	0.854	−0.607	−0.688	−0.798
PSS-10	−3.156 ***	−0.381	1.792 **	0.367	−1.364	−0.014
WHO-5	3.271 ***	0.095	−1.583	1.676	1.688	1.771
SWLS	0.854 **	0.095	−0.063	−0.081	0.792 *	0.014
CAS-PA	2.750 ***	0.524	−1.750 *	0.556	1.000	1.080
MBIGS	1.729	3.571 *	−1.313	−2.774	0.417	0.798
MBIGS_EE	0.271	1.119	−0.188	−1.866	0.083	−0.747
MBIGS_CY	−0.083	1.690 *	−0.313	−0.958	−0.396	0.732
MBIGS_PE	1.542 *	0.762	−0.813	0.051	0.729	0.813
UWES	4.833 **	0.095	−1.938	1.420	2.896	1.516
UWES_VIG	1.854 **	−0.286	−0.979	0.728	0.875	0.442
UWES_DEDI	1.229 *	−0.095	−0.625	0.473	0.604	0.378
UWES_ABS	1.750 **	0.476	−0.333	0.219	1.417	0.695

^a^ All the mean differences are statistically significant at *p* < 0.05. * *p* < 0.05, ** *p* < 0.01, *** *p* < 0.001.

**Table 4:** One-way ANOVA results for comparing all outcomes among groups at T2 and T3.

Outcomes		T2		*p*-Value	T3		*p*-Value
		95% Confidence Interval for Mean			95% Confidence Interval for Mean		
		Lower	Upper		Lower	Upper	
GAD-7				0.010 **			0.751
	Program	3.09	4.79		4.21	7.04	
	Control	4.63	8.08		4.35	7.57	
PHQ-9				0.002 **			0.162
	Program	3.37	4.76		3.93	5.91	
	Control	5.12	8.31		4.68	7.53	
PSS-10				0.001 ***			0.019 *
	Program	24.81	27.65		26.84	29.21	
	Control	28.33	31.67		28.73	32.00	
WHO-5				0.005 **			0.899
	Program	15.16	17.42		12.99	16.43	
	Control	11.29	15.09		13.02	16.71	
SWLS				0.354			0.314
	Program	12.54	13.51		12.38	13.54	
	Control	11.60	13.49		11.66	13.28	
CAS-PA				0.083			0.454
	Program	12.74	14.64		10.63	13.24	
	Control	10.58	13.70		11.11	14.29	
MBIGS				0.100			0.272
	Program	30.87	35.71		29.89	34.07	
	Control	33.02	41.12		30.46	38.14	
MBIGS_EE				0.013 *			0.196
	Program	8.01	10.49		7.36	10.77	
	Control	10.12	15.26		8.66	12.99	
MBIGS_CY				0.034 *			0.095
	Program	4.98	7.93		4.84	7.45	
	Control	7.04	11.30		6.03	10.39	
MBIGS_PE				0.060			0.237
	Program	15.93	19.24		14.99	18.55	
	Control	13.32	17.11		13.45	17.08	
UWES				0.001 ***			0.017 *
	Program	52.71	62.08		50.32	60.60	
	Control	40.83	50.60		42.71	51.56	
UWES_VIG				0.001 ***			0.031 *
	Program	18.57	21.97		17.49	21.10	
	Control	13.98	17.74		14.90	18.27	
UWES_DEDI				0.003 **			0.051
	Program	15.87	18.84		15.07	18.39	
	Control	12.22	15.68		12.79	16.06	
UWES_ABS				0.002 **			0.007 **
	Program	18.05	21.49		17.57	21.31	
	Control	14.29	17.52		14.64	17.60	

* *p* < 0.05, ** *p* < 0.01, *** *p* < 0.001.

## Data Availability

The data presented in this study are available on request from the corresponding author.

## Appendix A

**Table A1:** Description of the Activities for Each Sense.

Day	Activity	Sense Involved
1	Walking	Vision, hearing, smell, touch
2	Ecological photography	Vision, hearing
3	Ecological photography	Vision, hearing
4	Sketching butterflies	Vision, touch
5	Sketching butterflies	Vision, touch
6	Planting vegetables	Vision, hearing, smell, touch
7	Planting vegetables	Vision, hearing, smell, touch
8	Drinking herbal tea	Taste, smell, touch
9	Observing birds	Vision, hearing
10	Taking a nap in nature	Vison, hearing, smell, touch

**Table A2:** Descriptive statistics of the outcome variable at baseline.

	Program	Control	*p*-Value
	Mean (SD)	Mean (SD)	
Sample size	*n* = 48	*n* = 42	
Anxiety symptoms (GAD-7)	5.48 (3.242)	7.02 (5.362)	0.097
Depressive symptoms (PHQ-9)	5.60 (3.344)	6.90 (4.563)	0.124
Perceived stress (PSS-10)	29.39 (4.417)	30.38 (5.179)	0.328
Subjective well-being (WHO-5)	13.02 (4.215)	13.10 (5.617)	0.943
Satisfaction with life (SWLS)	12.17 (1.939)	12.45 (3.070)	0.594
Positive affect (CAS-PA)	10.94 (3.035)	11.62 (4.143)	0.372
Burnout (MBIGS)	31.56 (6.782)	33.50 (9.952)	0.278
Work Engagement (UWES)	52.56 (15.433)	45.62 (14.101)	0.029 *

* *p* < 0.05. GAD-7, 7-item Generalized Anxiety Disorder; PHQ-9, Patient Health Questionnaire-9; PSS-10, 10-item Perceived Stress Scale; WHO-5, WHO-Five Well-being Index; SWLS, Satisfaction with Life Scale; CAS-PA, 10-item positive affect subscale of Chinese Affect Scale; MBIGS, 14-item Maslach Burnout Inventory-General Survey; UWES, 17-item Utrecht Work Engagement Scale.
